# Biological therapies in moderate and severe psoriasis: perspectives and certainties

**Published:** 2014

**Authors:** MM Constantin, E Poenaru, T Constantin, C Poenaru, VL Purcarea, BR Mateescu

**Affiliations:** * ”Carol Davila” University of Medicine and Pharmacy, Bucharest, Romania; ** Cisco Systems Romania

**Keywords:** psoriasis, anti-TNF, immunogenicity

## Abstract

An inflammatory, proliferative condition with chronic evolution and systemic response, psoriasis, is positioned today among the most common inflammatory skin diseases affecting the Caucasian population worldwide.

With a significant incidence, psoriasis has been increasingly defined as a disease with a major impact on the patient's life and the society to which he/she belongs. This paper conducts an analysis of the currently available therapies for the treatment of moderate and severe psoriasis, therapies with biological agents obtained through sophisticated genetic engineering technologies. Recent research and the increasing interest in therapeutic methods as complete and efficient as possible make us optimistic and confident in the future.

**General consideration**

Psoriasis is an inflammatory, proliferative, immune-mediated disease, with chronic evolution and incidence between 2 and 4% in the general population [**1**]. Psoriasis vulgaris is the most common form of psoriasis and can be classified as it follows: type I characterized by an early onset (before 40 years old), positive family history and association with HLA- Cw6, HLA- DR7; type II defined by a late onset (over 40 years old), negative family history and no obvious associations with HLA.

Regarding the etiopathogenesis, although incompletely elucidated, a multifactorial and polygenic pattern of psoriasis that includes the presence of a number of susceptibility genes (PSORS 1-9) involved [**2**] has been described and also some disease-related genetic variations associated with tumor necrosis factor TNF- α, p40 subunit of interleukins 12/23 and IL 23 receptor. Many factors can trigger early events or contribute to disease exacerbation: stress, infections, smoking, certain medications such as beta-blockers, lithium salts, nonsteroidal anti-inflammatory drugs, etc. [**3**]. Psoriasis is the result of a complex immune skin reaction with a major inflammatory component involving elements of the innate and acquired (adaptive) immune system. All these are completed by an aberrant keratinocyte differentiation and proliferation. The activation of antigen-presenting cells results in the preferential development of T lymphocyte line with migration of the line and its proliferation (Th1, Th17) in the skin. It involves complex mechanisms dealing with a variety of surface receptors, chemotactic factors and mediators that orchestrate changes in psoriasis. The clinical examination of a patient with psoriasis vulgaris reveals typical erythematous-squamous lesions located on the extension areas of the extremities (elbows, knees), scalp, lesions that may be stable for a long period of time or may progress to a more intense disease manifestation, sometimes involving the entire body surface area. The association of the disease with various comorbidities such as metabolic syndrome, obesity, hyperglycemia, major cardiovascular diseases [**4**], and depressive syndrome accentuate the overall opinion with regard to the proven systemic response.

Psoriasis can develop in terms of body surface damage in three forms: mild (2% body surface area affected), moderate (2-10% surface affected) and severe (over 10%). Doctors today have many tools available to quantify the suffering of their patients. The PASI score (Psoriasis Area and Severity Index) assessing the extent and severity of psoriasis is the most commonly used in clinical trials [**5**]. A PASI score over 10 defines a moderate or severe disease, and PASI 75 and PASI 90 represent the dynamic parameters indicating the proportion of patients who achieved an improvement of 75% and 90% of the baseline score after treatment. Other measures commonly used to assess the severity of the disease are the following: PGA (Physician's Global Assessment) – general assessment of the physician; BSA (Body Surface Area) - body surface area affected; NAPSI (Nail Psoriasis Severity Index) – index showing nail psoriasis severity. The evaluation of the quality of life of patients with psoriasis is achieved through a series of questionnaires: Short Form - 36 (SF – 36), Dermatology Life Quality Index (DLQI), Psoriasis Quality of Life (PsoQoL), the most often used questionnaire being the DLQI [**6**].

Psoriasis causes a significant negative physical, psychosocial and economic impact on the patient [**7**]. Patients with psoriasis often have a low degree of satisfaction [**8**] and a poor compliance to the existing therapeutic approaches [**9**]. The explanation would be the limited efficacy, the poor compliance plus the fear and lack of sufficient information for the patient regarding the adverse effects of these therapies. Additionally, it seems that the doctor is often reluctant to administer systemic therapies [**10**], these implying an effort to monitor the side effects and sometimes involve risks due to the multiple interactions with other drugs [**11**]. In addition, there is a Romanian study, which indicates a progressive increase in the type, number, presentation forms of drugs available for the patients and health professionals [**12**].

**Systemic therapy with biological agents**

In this context, the modern biological therapies, obtained with the help of sophisticated genetic technologies in living organisms, are both a challenge and a promise of a more peaceful future for our patients [**13**].

Currently, there are 4 types of biological therapies available in Europe [**14**] and in Romania:

***1. Infliximab*** (Remicade), approved for psoriasis in September 2005, is a chimeric monoclonal antibody that is part of the family of TNF-α antagonists (increased affinity and specificity for TNF-α) with cytotoxic, inhibiting and neutralizing action in psoriasis and other inflammatory diseases which are based on the hyperproduction of TNF. By antagonizing TNF-α (increased in psoriasis), infliximab inhibits the release of proinflammatory cytokines and reduces the aberrant growth and proliferation of keratinocytes.

Infliximab is administered as IV infusion, 5 mg/kg of body weight for 2 hours at weeks 0, 2, 6 and afterwards every 8 weeks. A significant therapeutic response can be expected in 1-2 weeks. Clinical trials [**10**] showed a PASI 75 score obtained in 80% of the patients after 10 weeks of treatment and a PASI 90 score obtained in 50% of the patients for the same treatment period.

The common side effects are those caused by intravenous administration, infections, headache, fever, urticarial reactions, pruritus, occasionally tuberculosis, etc. The absolute contraindications include the following: active tuberculosis, significant active infection, chronic active hepatitis B, heart failure NYHA III/IV, hypersensitivity to infliximab, murine proteins or components, pregnancy or lactation.

Infliximab may be associated with methotrexate, especially since it was noticed that the methotrexate could reduce the incidence of development of autoantibodies to infliximab. Also, low doses of methotrexate are recommended especially when we have a significant joint damage. Infliximab (Remicade) is a biological agent sometimes preferred for its quickly installed action and proven effectiveness.

***2. Adalimumab*** (Humira), a drug approved for the treatment of psoriasis in December 2007, is a recombinant human immunoglobulin (IgG1) monoclonal antibody containing human peptide sequences.

Adalimumab binds specifically to TNF-α and neutralizes its biological function by blocking its interaction with TNF-α surface receptors: p55 and p75. It also modulates the TNF-induced and controlled biological effects. After a dose of 40 mg subcutaneously, the maximum serum concentration is 4.7 micrograms/dL, this being reached in 131 hours. The bioavailability of the drug is 64% [**15**].

The loading dose is of 80 mg followed by a dose of 40 mg and then 40 mg every two weeks, to provide a clinically significant response in a month. Efficacy studies revealed a PASI 75 at week 16 in 53-80% of patients for adalimumab and a PASI 100 in 14% of patients at week 16. On long term, 68% of the treated patients reach a PASI 75 at 60 weeks [**16**].

The common side effects of adalimumab are injection side reactions, upper respiratory tract infections, sinusitis, headache, skin rash; occasionally a tuberculosis outbreak can reactivate. Side effects are more serious in the elderly.

Patients receiving an anti-TNF should be evaluated for a potential risk of developing tuberculosis (detailed history, PPD TST, chest X-ray, Quantiferon test). If latent tuberculosis is suspected, the patient should receive prophylactic isoniazid for 9 months, this chemoprophylaxis starting one month before the administration of the biological therapy.

There are no conclusive studies regarding the combination of adalimumab with other therapies. An exception may be its combination with methotrexate, which seems to reduce the occurrence of autoantibodies (8% risk) [**17**].

***3. Etanercept*** (Enbrel) is a dimer of a chimeric protein resulting from the fusion of the extracellular binding domain of the p75 TNF-α receptor with the Fc fragment of human immunoglobulin IgG1. Etanercept inhibits TNF-α activity by competitive binding to proinflammatory cytokines, preventing the interaction with cell surface receptors. Approved for the treatment of psoriasis in September 2004, etanercept is slowly absorbed from the injection site, having a bioavailability of 60%, being metabolized and eliminated by excretion in the bile and urine.

The initial doses (0-12 W) are the following: 2 x 25mg or 2 x 50mg weekly and the maintenance doses: if PASI 75 is reached after 12W - 2 x 25mg; if PASI 75 is not achieved after 12W - 2 x 50mg until week 24. A significant clinical response can be obtained in 6-8 months. Efficacy studies [**14**,**18**] describe the achievement of PASI 75 at week 12, in 33% of the patients (2 x 25mg per week) and in 49% (2 x 50 mg). The substantial remission of injuries is also cited at week 24 (50%).

The vaccination with attenuated or live bacteria should be avoided, and, if absolutely necessary, the biological therapy should be discontinued 4-8 weeks before the immunization and resumed 2-3 weeks later. Screening is required to track down infections, tuberculosis, hepatitis or HIV infection and malignancies (lymphoma).

The therapies that etanercept may be combined with are those based on methotrexate and retinoids. Regarding the development of autoantibodies, etanercept is the biological drug with the lowest percentage in this respect (5%) [**19**,**20**].

***4. Ustekinumab*** (Stelara) is a biological agent newly registered (2009) for the treatment of moderate and severe psoriasis. IgG1k human monoclonal antibody, ustekinumab has an anti IL-12 and anti IL-23 action by binding to the p40 protein subunit (common to IL-12 and IL-23) [**21**]. The efficacy studies describe a PASI 75 score at week 12, in 67% of the patients treated with 45 mg and in 73% of those receiving 90 mg ustekinumab [**22**]. The therapy with this biological agent is tailored to the individual (dosage based on weight), with an increased adhesion of this agent (given at 12 weeks), an excellent tolerability and maintenance of the effect on the long term.

## Conclusions

The choice of the TNF antagonist is based on the clinical needs and risk assessment of each patient. Psoriasis is a chronic pathological, severe, multi-systemic condition with whimsical evolution and a significant negative impact on the patient’s quality of life.

Lately, the global economy has been affected by the financial crisis. However, in Romania the pharmaceutical market has been evolving similar to the other European countries, in spite of our legislation instability, lack of transparency and ineffective communication [**23**].

Biological therapy, recently involved in the treatment of psoriasis, is innovative, selective and specific, tailored to the treatment and highly effective, reintegrating patients to society and restoring their dignity.
